# Solution Structural Analysis of the Single-Domain Parvulin TbPin1

**DOI:** 10.1371/journal.pone.0043017

**Published:** 2012-08-10

**Authors:** Lifang Sun, Xueji Wu, Yu Peng, Jian Yuan Goh, Yih-Cherng Liou, Donghai Lin, Yufen Zhao

**Affiliations:** 1 The Key Laboratory of Chemical Biology of Fujian Province, College of Chemistry and Chemical Engineering, Xiamen University, Xiamen, China; 2 NMR Laboratory, Shanghai Institute of Materia Medica, Chinese Academy of Sciences, Shanghai, China; 3 NUS Graduate School for Integrative Sciences and Engineering, National University of Singapore, Singapore; 4 Department of Biological Sciences, National University of Singapore, Singapore; National Institute for Medical Research, Medical Research Council London, United Kingdom

## Abstract

**Background:**

Pin1-type parvulins are phosphorylation-dependent peptidyl-prolyl *cis-trans* isomerases. Their functions have been widely reported to be involved in a variety of cellular responses or processes, such as cell division, transcription, and apoptosis, as well as in human diseases including Alzheimer's disease and cancers. TbPin1 was identified as a novel class of Pin1-type parvulins from *Trypanosoma brucei*, containing a unique PPIase domain, which can catalyze the isomerization of phosphorylated Ser/Thr-Pro peptide bond.

**Methodology/Principal Findings:**

We determined the solution structure of TbPin1 and performed ^15^N relaxation measurements to analyze its backbone dynamics using multi-dimensional heteronuclear NMR spectroscopy. The average RMSD values of the 20 lowest energy structures are 0.50±0.05 Å for backbone heavy atoms and 0.85±0.08 Å for all heavy atoms. TbPin1 adopts the typical catalytic tertiary structure of Pin1-type parvulins, which comprises a globular fold with a four-stranded anti-parallel β-sheet core surrounded by three α-helices and one 3_10_-helix. The global structure of TbPin1 is relatively rigid except the active site. The 2D EXSY spectra illustrate that TbPin1 possesses a phosphorylation-dependent PPIase activity. The binding sites of TbPin1 for a phosphorylated peptide substrate {SSYFSG[p]TPLEDDSD} were determined by the chemical shift perturbation approach. Residues Ser15, Arg18, Asn19, Val21, Ser22, Val32, Gly66, Ser67, Met83, Asp105 and Gly107 are involved in substantial contact with the substrate.

**Conclusions/Significance:**

The solution structure of TbPin1 and the binding sites of the phosphorylated peptide substrate on TbPin1 were determined. The work is helpful for further understanding the molecular basis of the substrate specificity for Pin1-type parvulin family and enzyme catalysis.

## Introduction

The peptidyl-prolyl bond can exist in two distinct *cis* or *trans* conformations, due to the unique side chain of proline, which in turn affects an overall protein structure. When the peptide bond switches between *cis-trans* conformation, protein structures will remodel accordingly, generating functional proteins [1]–[5], although the structural interconversion between the *cis* and *trans* conformations is substantially slow and a rate-limiting step. Interestingly, a family of peptidyl-prolyl *cis-trans* isomerases (PPIases) was identified, which all possessed a PPIase activity and could catalyze the intrinsically slow *cis-trans* isomerization [2], [5], [6]. So far, the PPIases family is suggested to be divided into three structurally distinct subfamilies: cyclophilins (Cyps), FK506-binding proteins (FKBPs), parvulins [2], [5], [7], [8].

In terms of substrate specificity, the parvulin subfamily can be further divided into the Pin1-type parvulins (phosphorylation-dependent) and the non Pin1-type parvulins (phosphorylation-independent) [2], [8]–[12]. The Pin1-type parvulins specifically catalyze the *cis-trans* isomerization of either phosphoserine- or phosphothreonine-proline (pSer/pThr-Pro) peptide bond. The phosphorylation-dependent isomerization is unique among all PPIases [13]. The phosphorylation specificity indicates that Pin1 plays an important role in the regulation of proline-directed phosphorylation associated signaling pathways [14], [15] [15]. For example, protein kinases such as MAP kinase (MAPK) and CDK2 specifically phosphorylate the *trans* conformation of Ser/Thr-Pro peptide bond [16], [17]. Phosphorylation of the substrates would further slow down the *cis-trans* interconversion rate. However, Pin1-type parvulins could accelerate the interconversion process. Evidences for the biological importance of the Pin1-type parvulins have been elucidated [3], [17]–[24].

Based on the structure, two distinct classes of Pin1-type parvulins have been identified in various organisms. Most of Pin1-type parvulins such as human Pin1 (hPin1), yeast ESS1/PTF1, and Drosophila Dodo, consist of two domains: an N-terminal WW domain and a conserved C-terminal catalytic PPIase domain. The WW domain is a binding module that specifically recognizes pSer/pThr-Pro motifs, while the C-terminal PPIase domain catalyzes the isomerization of pSer/pThr-Pro prolyl-peptide bonds [25]–[28]. However, several Pin1-type parvulins lacking of the WW domain have been reported, such as plant Pin1s [29]. Recently, two new members of the parvulin subfamily, TbPin1 and TbPar42, were identified from *Trypanosoma brucei* [10], [30]. Like plant Pin1s, TbPin1 lacks the N-terminal WW domain and only contains the catalytic PPIase domain. Instead of a WW domain, TbPar42 contains a forkhead-associated (FHA) domain at its N-terminal and a well conserved PPIase domain at C-terminal. TbPin1 has an ability to compensate for the loss of Ess1 function in yeast but TbPar42 lacks the ability [30], indicating that TbPin1 is a Pin1-type parvulin and TbPar42 is a non Pin1-type parvulin. Furthermore, studies of their subcellular localization in *Trypanosoma brucei* showed that TbPin1 was uniformly distributed in the cytoplasm, while TbPar42 was localized in the nucleus [30]. On the other hand, previous studies demonstrated that hPin1 was localized in both the nucleus and cytoplasm, and that its nuclear localization was due to the interaction of the WW domain with the target protein [20], [24], [31]. Thus, it is speculated that the exclusion of TbPin1 from the nucleus might be due to its lack of the WW domain. However, studies on plant Pin1s (such as DlPar13 and LjPar1) have shown that despite lacking the WW domain, plant Pin1s could still be localized in the cytoplasm and nucleus [26], [32]. These studies implicated that other structural or physiological conditions might play crucial roles in the specific localization of parvulins, and that TbPin1 potentially have some functions different from its homologues in other species.

The detailed structural interpretations of parvulins provide valuable information for addressing the functions of parvulins. So far, the three-dimensional structure of TbPin1 has not been interpreted and characterized. In the present work, we determined the solution structure and dynamics of TbPin1, performed the PPIase activity analysis and chemical shift perturbation for TbPin1 using NMR spectroscopy.

## Results

### Sequence alignment of TbPin1 with selected members of the parvulin family

**Figure 1:**
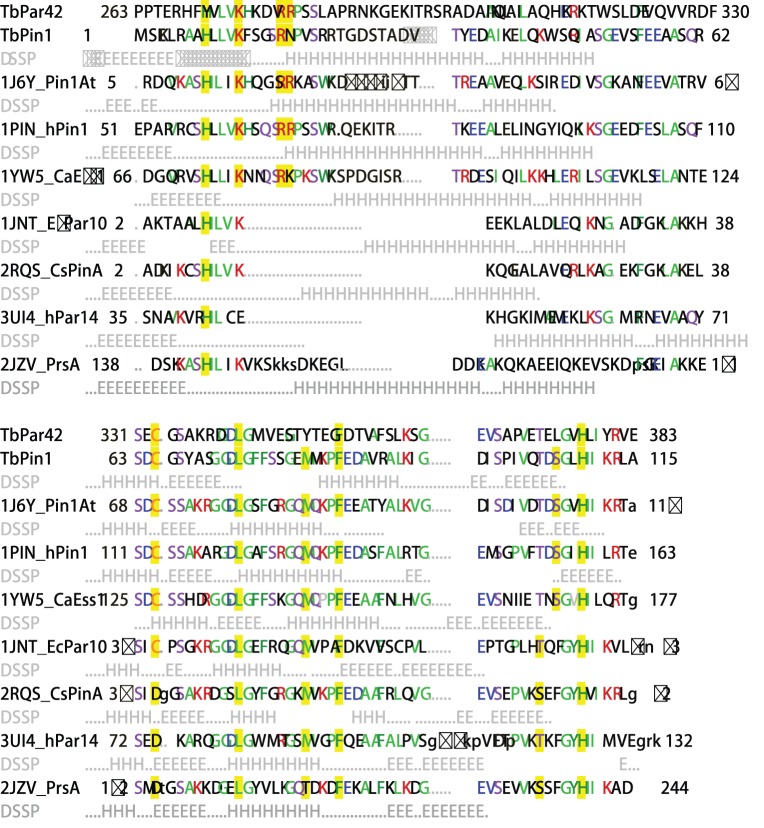
Sequence alignment of TbPin1 with other parvulins. The following structures and protein sequences were obtained from PDB and SwissProt: TbPin1 (Q57YG1); TbPar42 (Q57XM6); 1J6Y, Pin1At (Q9SL42); 1PIN, hPin1 (Q13526); 1YW5, CaEss1 (G1UA02); 1JNT, EcPar10 (P0A9L5); 2RQS, CsPinA (O74049); 3UI4, hPar14 (Q9Y237); 2JZV, PrsA (P60747). The sequences of parvulins were aligned with that of TbPin1 and the available tertiary structures of parvulins were also aligned onto the structure of TbPin1 using DaliLite [33]. DSSP information for helix (H) and strand (E) conformations from the PDB files is given in light gray below the protein sequences except TbPar42. Aligned residues are written as capital letters and the most frequent residues are colored in each column. Residues considered crucial for the PPIase activity are highlighted in yellow.

To understand the primary sequence relationship, the amino acid sequences of TbPin1 (Swiss-Prot ID: Q57YG1) and the PPIase domain of TbPar42 (Q57XM6) were aligned with those of selected Pin1-type parvulins including Pin1At (Q9SL42) from *A. thaliana*; hPin1 (Q13526) from *H. sapiens* and CaEss1 (G1UA02) from *C. albicans*, and some non Pin1-type parvulins including EcPar10 (P0A9L5) from *E. coli*; CsPinA (P60747) from *C. symbiosum*; hPar10 (Q13526) from *H. sapiens*; PrsA-PPIase domain (P60747) from *S. aureus*. Furthermore, the available structures of the parvulins were also aligned onto the structure of TbPin1 using DaliLite [33]. Sequence alignment shows that the catalytic PPIase domain is well conserved. The subfamilies of parvulins differ in length and composition of the β1/α1 loop (Figure 1). In Pin1-type parvulins, the loop is considered the phosphate binding loop containing several positively charged residues (Lys63, Arg68 and Arg69 in hPin1) [25]. It is thereby expected that the loop could induce the preference for substrates with a negatively charged residue especially the phospho-Ser/Thr [25], [34]. In non Pin1-type parvulins, the β1/α1 loop is mostly missed or shorter than that in Pin1-type parvulins [34]–[38], such as EcPar10, CsPinA, hPar14 and PrsA-PPIase domain (Figure 1). However, the loop in TbPar42 is longer than those in other parvulins, which also contains a high number of positively charged residues (Lys276, Arg280 and Arg281) similar to those in Pin1-type parvulins. Thus, the role of the β1/α1 loop both in Pin1-type parvulins and in non Pin1-type parvulins is in need of further investigation.

### Chemical Shift Assignments of TbPin1

**Figure 2:**
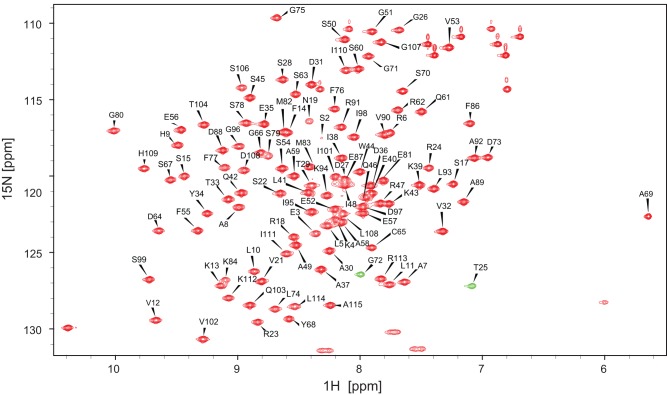
Assigned ^1^H-^15^N HSQC spectrum of TbPin1 in 20 mM sodium phosphate buffer at pH 7.0. The spectrum was recorded at 298 K on a Varian Unity Inova 600 MHz spectrometer. Resonance assignments of backbone amide groups are indicated by the residue type and number. The unlabeled resonances are the side chain amides. T25 and G72 are shown in negative resonances corresponding to the resonances aliased in the ^15^N dimension. Positive resonances are colored red and negative resonances are colored green.

Sequence-specific assignments of backbone ^1^H, ^13^C and ^15^N resonances of TbPin1 were obtained based on a suite of 3D heteronuclear NMR spectra including HNCACB, CBCA(CO)NH, HNCA, HN(CO)CA, HNCO, HN(CA)CO spectra. Side-chain assignments of ^1^H and ^13^C resonances of TbPin1 were achieved on the basis of 3D HBHA(CO)NH, H(CCH)(CO)NHTOCSY, (H)CC(CO)NHTOCSY, HCCH-TOCSY, CCHTOCSY and ^15^N-edited TOCSY spectra. More than 95.9% of the backbone assignments were obtained and about 85.7% of the side chain assignments were extracted by this procedure with the exception of signals from aromatic side chains and three residual residues (Gly-Ser-His) from the N-terminal extension. All assigned ^1^H, ^13^C and ^15^N chemical shifts of TbPin1 were deposited in the Biological Magnetic Resonances Bank (BMRB ID: 17918), and the assigned ^1^H-^15^N HSQC spectrum is shown in Figure 2.

### NMR structures of TbPin1 in solution

**Figure 3:**
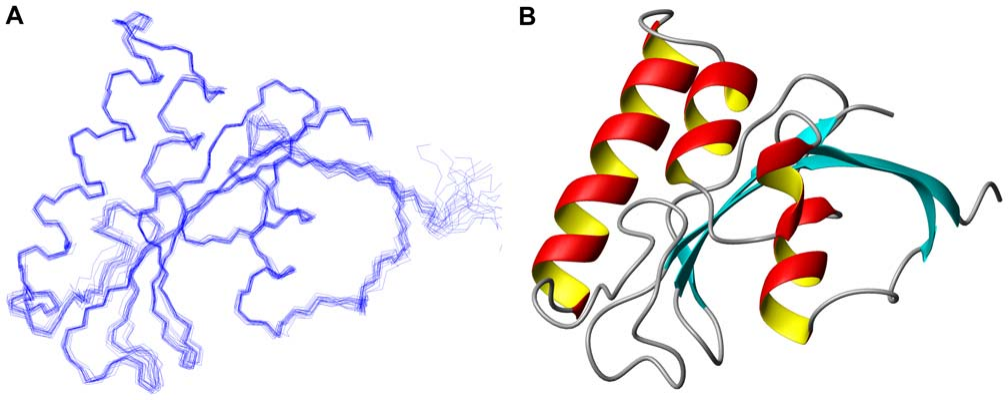
Solution structure of TbPin1. (A) Superimposed backbone traces of the 20 lowest energy structures in the structure ensemble; (B) Ribbon diagram of the mean structure showing the secondary structure elements. The structure was determined in 20 mM sodium phosphate buffer at pH 7.0. Structure visualization is generated using MOLMOL.

Using the Aria2.2/CNS1.2 software [39], [40], we calculated the solution structure of TbPin1 with 1918 distance restraints (711 intra-residual, 453 sequential, 195 short-range, 103 medium-range and 456 long-range NOEs) derived from 3D ^15^N-edited and ^13^C-edited NOESY-HSQC spectra, and 187 backbone torsion angle restraints generated by TALOS+ [41]. A family of 200 structures was calculated according to the simulated annealing protocol. Then, 20 lowest energy structures were selected and further refined in water. The structural statistics of the final 20 lowest energy conformers are summarized in Table 1. There are no distance restraint violations greater than 0.5 Å and no torsion angle restraint violations above 5°in the 20 lowest energy structures (Figure 3A). Ramachandran plot analysis of the 20 lowest energy structures indicates 99.1% of residues are located in the allowed regions. The average RMSD values are 0.50±0.05 Å for backbone heavy atoms and 0.85±0.08 Å for all heavy atoms. The atomic coordinates of TbPin1 were deposited in the Protein Data Bank (PDB: 2LJ4). The three-dimensional structure of TbPin1 is a typical Pin1-type parvulin-fold consisting of a four-stranded anti-parallel β-sheet core (β1, residues 4–12; β2, 73–78; β3, 101–103; β4, 108–113), three α-helices (α1, 34–50; α2, 55–62; α3, 84–90) and one 3_10_-helix (η1, 66–70) surrounding the core (Figure 3B).

**Table 1:** Structural statistic for the 20 lowest energy structures of TbPin1.

Restraints for calculations	
Total NOE restraints used	1918
Intraresidue (|i–j| = 0)	711
Sequential (|i–j| = 1)	453
Short-range (2≤|i–j|≤3)	195
Medium-range (4≤ |i–j|≤5)	103
Long-range (|i–j|>5)	456
**Hydrogen bond restraints**	35
**Dihedral angle restraints (Φ and Ψ)** a	187

Restraints violations (20 structures)	
NOE distance violation (>0.5Å)	0
NOE distance violation (>0.3Å)	0.5±0.50
NOE distance violation (>0.1Å)	42.40±4.35
Torsion angle violation (>5°)	0

RMSDs from mean structure (Å)	
All residues (backbone)	0.50±0.10
Secondary structures (backbone)	0.22±0.04
All residues (heavy atoms)	0.85±0.08
Secondary structures (heavy atoms)	0.50±0.05

Ramachandran plot (PROCHECK)^b^	
Residues in most favorable regions (%)	85.2
Residues in additional allowed regions (%)	12.7
Residues in generously allowed regions (%)	1.2
Residues in disallowed regions (%)	0.9

a Dihedral angle restraints are generated by TALOS+ [41].

b Quality of the ensemble of the 20 lowest-energy structures of TbPin1 was assessed by PROCHECK-NMR [61].

In TbPin1, hydrophobic residues (Leu74, Met82 and Phe86) and two histidine residues (His9 and His109) forming the hydrophobic core are located on the concave side of the β-sheet cluster. The histidine side chains adopt the very similar orientation observed in other parvulin structures. However, the imidazole moieties show different ring-flipping states in comparison with those in hPin1 (PDB: 1PIN, 1F8A) and the orientations reported in hPar14 and PrsA-PPIase domain [34], [42]. It may be due to that the limited resolution disallows an unequivocal assignment.

**Figure 4:**
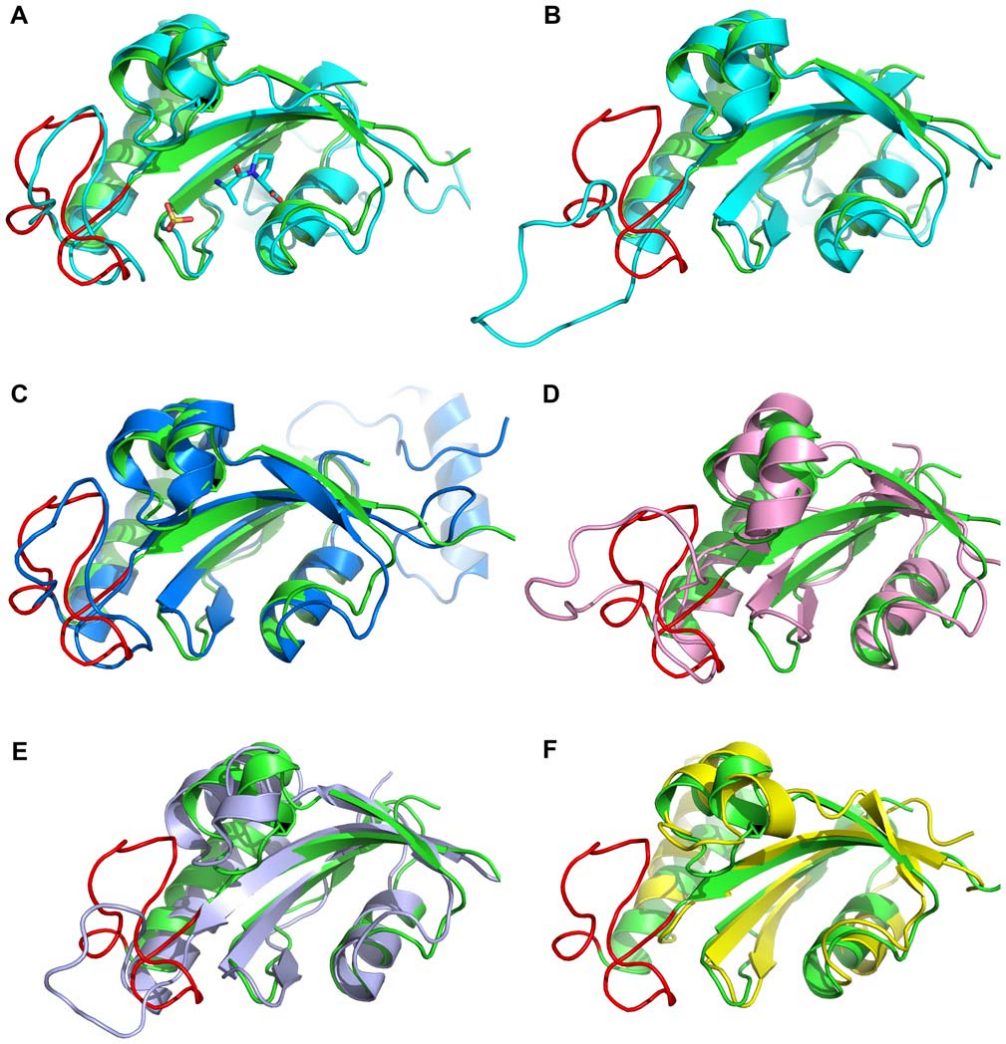
Structural comparison of TbPin1 (2LJ4) with other parvulins. (A) hPin1 in complex with the Ala-Pro dipeptide and a sulfate ion (1PIN); (B) hPin1 without a sulfate ion (1F8A); (C) CaEss1 (1YW5); (D) Pin1At (1J6Y); (E) PrsA-PPIase domain (2JZV); (F) EcPar10 (1JNT). The structures of TbPin1, hPin1, CaEss1, Pin1At, EcPar10 and PrsA-PPIase domain are colored green, cyan, blue, pink, lightblue and yellow, respectively. The β1/α1 loop of TbPin1 is displayed in red. The sulfate ion is used to mimic to the phosphate group.

Superposition of backbone heavy atoms of PPIase domains of hPin1 (PDB: 1PIN), CaEss1 (PDB: 1YW5), AtPin1 (PDB: 1J6Y), EcPar10 (PDB: 1JNT) and PrsA (PDB: 2JZV) with TbPin1 show RMSD values of 1.6 Å, 1.8 Å, 2.6 Å, 2.4 Å, 1.7 Å, respectively (Figure 4). They display a well conserved PPIase domain. In the solution structure of TbPin1, the β1/α1 loop (residues 14–33) exhibits a folded conformation, which has also been observed in hPin1 (PDB: 1PIN), Pin1At (PDB: 1J6Y) and CaEss1 (PDB: 1YW5) (Figure 4). In the crystal structure of hPin1 (PDB: 1PIN), a sulfate ion was found to be bound with the β1/α1 loop [25] (Figure 4A). In the solution structure of Pin1At, sodium sulfate salt was employed to stabilize the β1/α1 loop [43], and a buffer contained phosphate was used to crystallize CaEss1 [27]. Moreover, Daum et al. suggested the phosphate group in the buffer could be bound to the phosphate binding pocket [44]. On the other hand, the β1/α1 loop in the crystal structure of hPin1 (PDB: 1F8A) shows an unfolded conformation (Figure 4B), in which neither phosphate nor sulfate was present [45]. Therefore, a phosphate group would be bound to the phosphate binding pocket to induce the folded conformation of the β1/α1 loop in TbPin1, which was dissolved in 20 mM sodium phosphate buffer. In addition, structural alignment of the two non Pin1-type parvulins (PrsA-PPIase domain and EcPar10) with TbPin1 demonstrates that the β1/α1 loop of PrsA-PPIase domain is shorter than that of TbPin1 (Figure 4E), and the loop of EcPar10 is totally absent (Figure 4F). Thus, this loop might be important for the phosphorylation-dependent *cis-trans* isomerization, and potentially serves for distinguishing Pin1-type parvulins from non Pin1-type parvulins.

### Enzymes activity analysis

**Figure 5:**
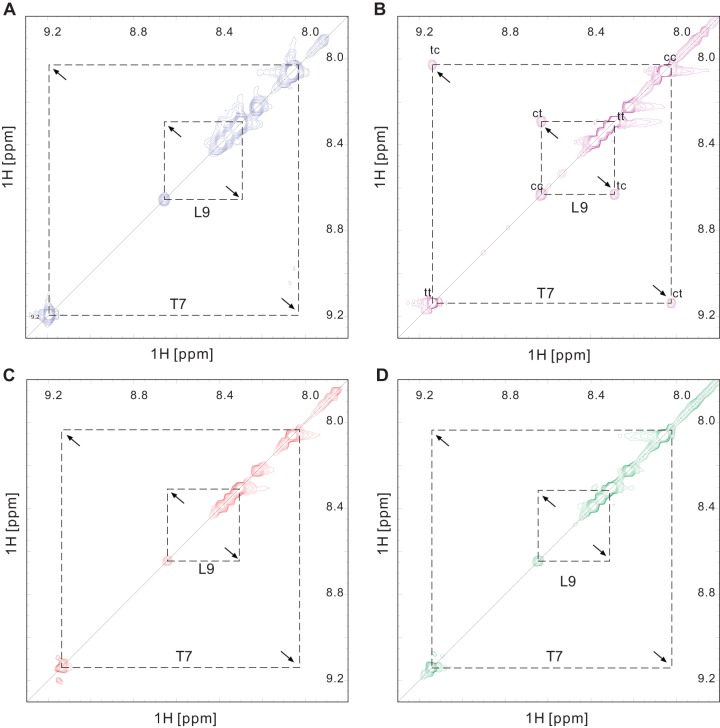
TbPin1 catalyzes the *cis-trans* isomerization of a phosphorylated peptide. A selected region of the 2D-EXSY spectrum of the phosphorylated peptide SSYFSG[p]TPLEDDSD is displayed (A) in the absence of TbPin1; (B) in the presence of TbPin1; (C) in the presence of the TbPin1-C65A mutant; (D) in the presence of the FHA-truncated TbPar42 mutant (TbPPIase). A mixing time of 300 ms was used. The *cis* and *trans* T7-HN and L9-HN are shown in the spectra, which were assigned based on the 2D-TOCSY spectrum of the peptide. Diagonal peaks from *cis* and *trans* conformers are indicated by cc and tt, respectively, whereas exchange peaks resulting from isomerization are labeled with ct and tc.

Based on a phosphorylated peptide {SSYFSG[p]TPLEDDSD} derived from the substrate of Pin1At, Agamous-like 24 (AGL24) [22], we performed isomerase activity assays in vitro for TbPin1 using 2D ^1^H-^1^H NMR spectra including EXSY (exchange spectroscopy) and TOCSY (total correlation spectroscopy) spectra. The pSer/pThr-Pro motif displays two distinct sets of ^1^H signals in the 2D TOCSY and 2D EXSY spectra. No cross-peaks between the *cis* and *trans* isomers could be observed in the 2D EXSY spectrum due to the slow exchange rate between the *cis* and *trans* conformations [22], [46], [47]. Thus, in the absence of TbPin1, no cross peaks were observed in the EXSY spectrum of the phosphorylated peptide (Figure 5A), indicating that the exchange between the *cis* and *trans* conformations was too slow to be detected on the NMR timescale. By contrast, in the presence of TbPin1, the proline isomerization rate of the phosphorylated peptide was greatly enhanced, and cross-peaks resulting from the conformational exchange were observed in the EXSY spectrum (Figure 5B). In the presence of the TbPin1-C65A mutant, no cross-peaks were detected (Figure 5C), hence the PPIase activity to the peptide was shut down by the mutation. Furthermore, as shown in Figure 5D, no cross-peaks were observed in the presence of the FHA-truncated TbPar42 (TbPPIase) mutant. In addition, no exchange peaks were observed for the non-phosphorylated peptide SSYFSGTPLEDDSD in the presence of and absence of TbPin1, or TbPPIase (Figure S1 in supporting information). These results suggest that TbPin1 is a Pin1-type parvulin possessing phosphorylation-dependent isomerase activity. Shut down the activity by mutation of Cys65 to Ala further confirms that the conserved Cys65 residue plays a crucial role in the PPIase activity of TbPin1, as observed for other Pin1-type PPIases [25], [29].

### Determination of substrate binding sites on TbPin1

**Figure 6:**
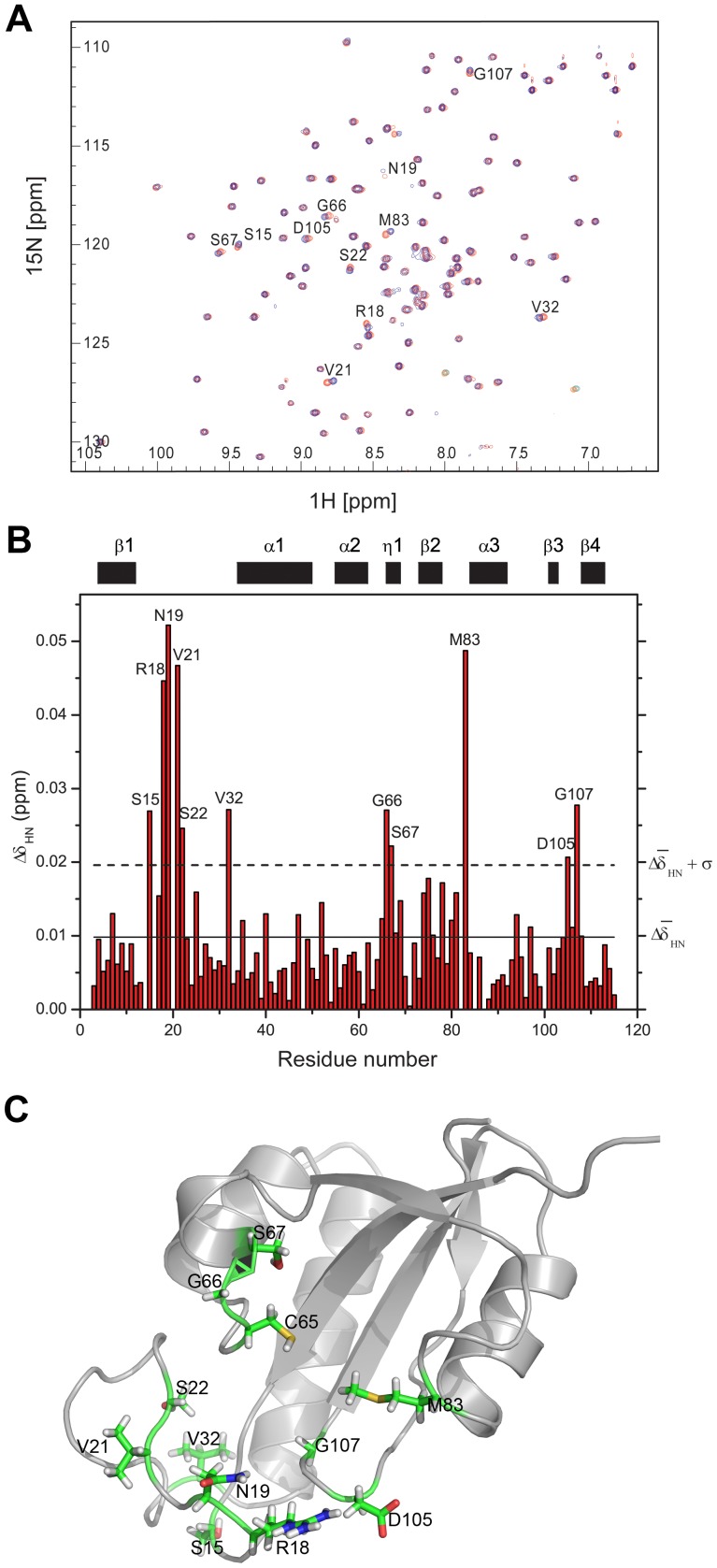
Chemical shift perturbation analysis reveals the substrate binding pocket on TbPin1. (A) Overlay of the ^1^H-^15^N HSQC spectra of TbPin1 free (red) and in complex with the unlabeled phosphorylated peptide substrate (blue). The molar ratio of TbPin1 to the phosphorylated peptide is 1∶8. (B) Diagram of amide chemical shift changes (Δδ_HN_) of TbPin1 versus residue number at a molar ratio of 1∶8. The average amide chemical shift change (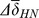) and the mean standard deviation (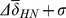) are indicated with solid and dashed lines, respectively. Residues with chemical shift changes larger than 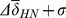 (dashed line) are considered to be involved in substantial contact with the substrate. (C) Mapping the substantial contact residues on the tertiary structure of TbPin1. Residues with significant chemical shift change are highlighted in stick style.

Chemical shift perturbation experiments were used to determine the binding sites of the phosphorylated peptide {SSYFSG[p]TPLEDDSD} on TbPin1. ^15^N-labeled TbPin1 was titrated stepwise with the peptide to a final molar ratio of 1∶8. 2D ^1^H-^15^N HSQC spectra were acquired following each addition of the peptide. To some extent, the peptide titration caused non-significant chemical shift perturbations in the HSQC spectra of TbPin1. The dissociate constant K_d_ was estimated to be about millimolar. Figure 6A shows the HSQC spectrum of unliganded TbPin1 protein (red peaks) overlaid with the final HSQC spectrum in which TbPin1 was saturated with the peptide (blue peaks). To identify the residues involved in the interaction of TbPin1 with the peptide, the magnitudes of the chemical shift changes caused by the peptide binding versus residue number are shown in Figure 6B. Residues on TbPin1 exhibiting significant amide chemical shift changes (>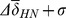) are mapped to the tertiary structure of TbPin1 (Figure 6C). Approximately 11 peaks are observed with significant chemical shift perturbations. These residues are concentrated to the catalytic loop (Ser15, Arg18, Asn19, Val21, Ser22 and Val32), η1 (Gly66 and Ser67), and the linker regions connecting β2 and α3 (Met83), β3 and β4 (Asp105 and Gly107).

### Dynamics in the free enzyme

**Figure 7:**
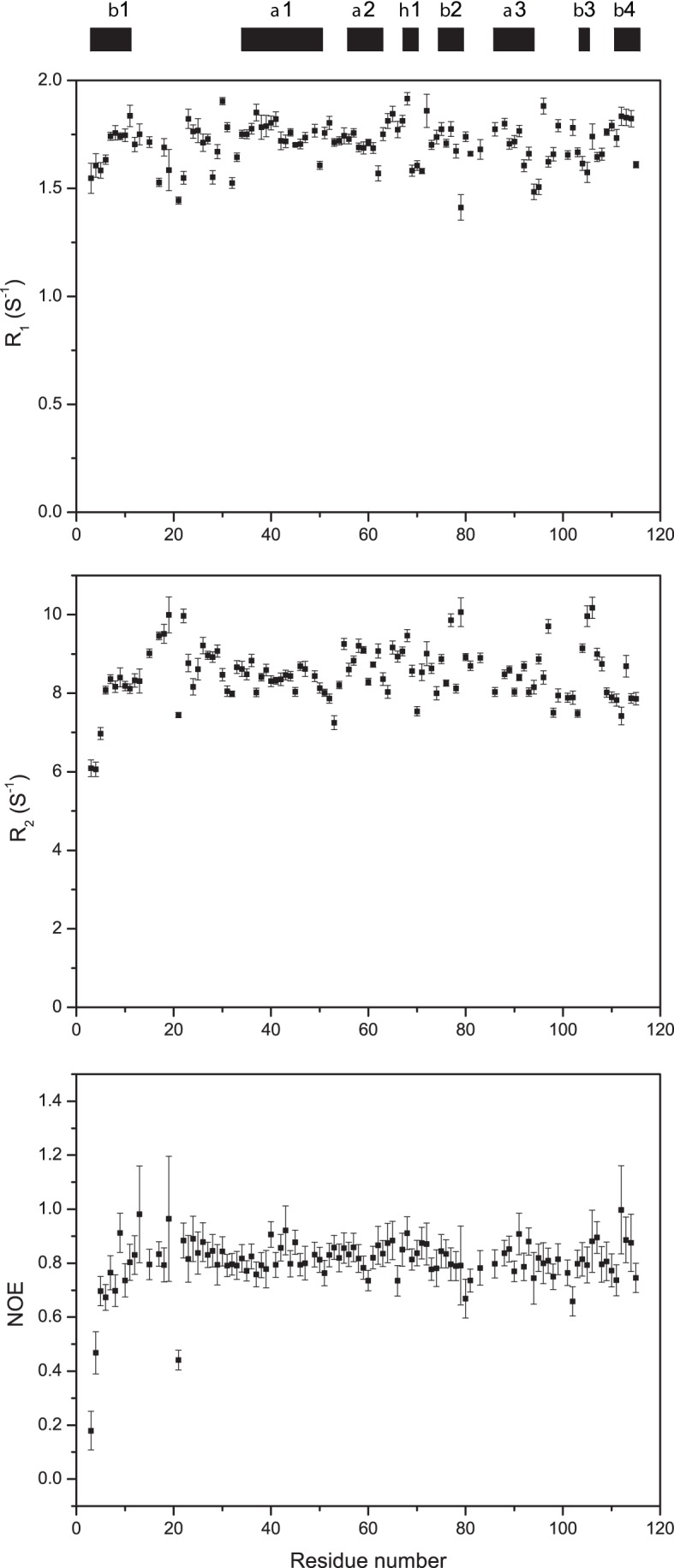
Relaxation rates R_1_, R_2_ and {^1^H}-^15^N heteronuclear NOEs of TbPin1. The regular secondary structure elements are indicated on the top. NMR spectra were recorded at 298 K on a Varian Unity Inova 600 MHz spectrometer. Proteins were dissolved in 20 mM phosphate sodium buffer containing 1 mM DTT, 0.1 mM NaN_3_, 10% D_2_O, pH 7.0.

To analyze the global and local backbone dynamics of TbPin1, we performed ^15^N relaxation experiments. As a whole, 105 assigned residues were used except the unobservable resonances and partially overlapped peaks such as Phe14 and Met82. The relaxation rates R_1_, R_2_ and heteronuclear NOEs versus residue number are shown in Figure 7. The R_1_ values do not change markedly with the sequence, ranging from 1.4 to 1.9 s^−1^. Different from the R_1_ distribution, the R_2_ values are relatively variable with residue number, ranging from 6.0 to 10.2 s^−1^. The residue Ser106 shows the largest R_2_ value over 10.1 s^−1^, and Asn19, Ser22 also display distinctly large R_2_ value as high as 10.0 s^−1^. Almost all of the NOE values are between 0.7 and 0.9 except for Glu3, Lys4 and Val21 (< 0.6), indicating that the overall structure of TbPin1 is highly rigid.

### Analysis of ^15^N relaxation data

**Figure 8:**
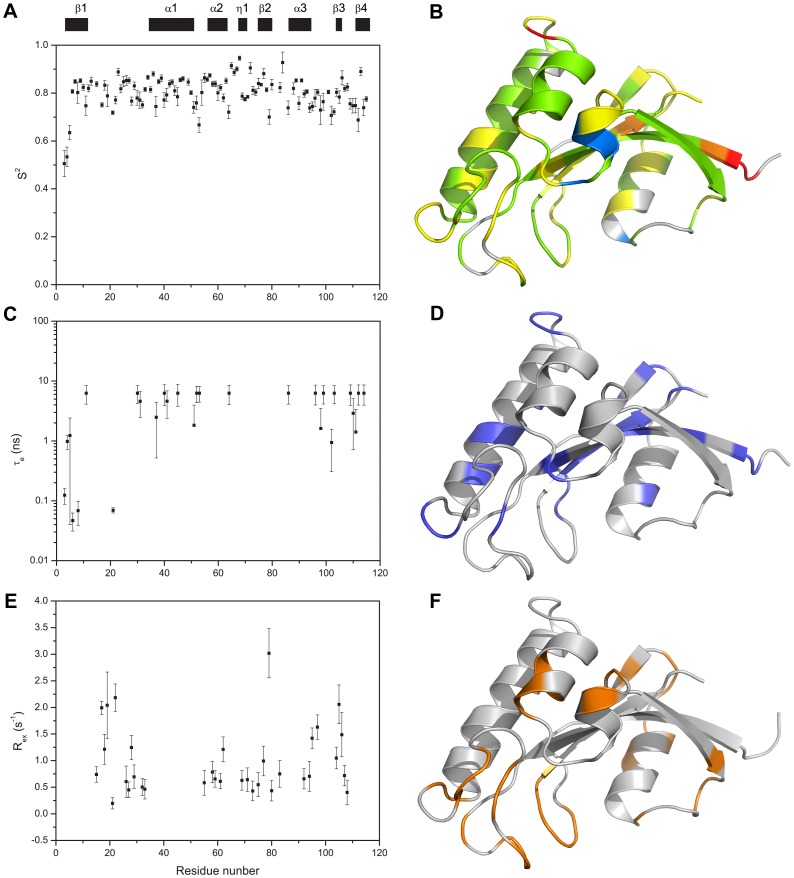
Model-free analysis of TbPin1. (A, C and E) The plots of the order parameters S^2^, the effective internal correlation time τ_e_, and the conformational exchange rate R_ex_ versus residue number of TbPin1, respectively; (B, D and F) Residues with S^2^, τ_e_ (blue) and R_ex_ (orange) values are mapped onto the solution structure of TbPin1, respectively. (B) Gray, S^2^ unavailable due to the absence of NMR data or failure in NMR data fitting; red, S^2^<0.6; orange, 0.6≤S^2^<0.7; yellow, 0.7≤S^2^<0.8; green, 0.8≤S^2^<0.9; and blue, 0.9≤S^2^<1.0. The ribbon graph was generated by PyMOL.

We used both the TENSOR2 program [48] and Mathematica notebooks from Spyracopoulos' lab [49] to analyze the backbone ^15^N relaxation data for TbPin1. Residues were eliminated due to NOE <0.65 and filtered due to short T_2_ values based on the criterion {[(<T_2_>–T_2n_)/<T_2_>–(<T_1_>–T_1n_)/<T_1_>] >1.5 SD}, where T_2n_ is the T_2_ value of residue n, and <T_2_> is the average T_2_ value, SD is the standard deviation of (<T_2_>–T_2n_)/<T_2_>–(<T_1_>–T_1n_)/<T_1_> [50], [51]. Thus, 78 out of 105 residues were used to determine the rotational diffusion parameters of TbPin1. The inertia tensor of the TbPin1 protein was calculated from the PDB coordinates. Its principal value ratios (I_x_: I_y_: I_z_) were 1.00:0.89:0.56, allowing the approximation of the protein molecule as a prolate ellipsoid. The D_∥_: D_⊥_ ratio of the rotational diffusion tensor of TbPin1 was calculated to be 1.17±0.01, suggesting that the axially symmetric model was suitable for the data fitting. In the model-free approach, mobility was characterized by the order parameter S^2^, which could be interpreted as the amplitude of the inter-motion on a nanosecond time scale. The calculated S^2^ values versus residue number are mapped into the tertiary structure of TbPin1 with a color pattern (Figure 8). Residues with S^2^ values ranging from 0.7 to 0.9 are located on most of secondary structure elements, indicating that TbPin1 adopts a much rigid structure. Only Cys65, Ser67, Tyr68, Gly72 and Lys84 show relatively high S^2^ value (S^2^>0.9), indicating that this region (Figure 8B) is very restricted. The other two dynamics parameters, i.e., correlation time (τ_e_) for ps-ns timescale internal motion, and conformational exchange rate (R_ex_) for µs-ms timescale internal motion are shown in Figure 8. Residues with R_ex_ >1.0 S^−1^ are also mostly located on the region of the peptide binding pocket, including the catalytic loop (Ser15, Arg18, Asn19, Ser22 and Ser28), the α2 helix (Arg62), the C-terminal end of β2 (Phe77 and Ser79), the linker regions connecting α3 and β3 (Ile95 and Asp97), β3 and β4 (Thr104, Asp105, Ser106) (Figure 8). This result indicates that TbPin1 is well structured except the binding pocket of the phosphorylated peptide which exhibits significant conformational exchange.

## Discussion

In this work, we have determined the solution structure of the Pin1-type parvulin TbPin1 from *Trypanosoma brucei*. TbPin1 has the unique PPIase domain with a lack of the WW domain, which shares high homologies with other members of Pin1-type parvulins, and exhibits phosphorylation-dependent PPIase activity (Figure 1 and 5).

Recent studies of hPin1 and other parvulins have provided new insights into the functional status of active site residues [34], [42], [52], [53]. In the Pin1-type parvulin family, the basic cluster (Lys63, Arg68 and Arg69 in hPin1) has been considered essential for phospho-specific recognition [25], [54]. However, Behrsin et al. used a unigenic evolution strategy to reveal that only 19 residues were perfectly conserved in the hPin1 sequence, and the two residues crucial for the phosphate binding, Arg68 and Arg69, were not always conserved through unigenic evolution [52]. They proposed that only one basic residue (Arg) at either position 68 or 69 was essential for the function of hPin1. In line with this finding, alignment of TbPin1 with its homologues shows that the two Arg residues at the positions are not all conserved. Arg69 in hPin1 is replaced by Asn19 in TbPin1, in the case of CaEss1 the equivalent basic residue is Lys83 (Figure 1). Interestingly, TbPar42 shows absolutely conserved residues Lys276, Arg280, Arg281 in the corresponding position. Thereby, TbPar42 would have a phosphate-binding loop or prefer the substrates with a negatively charged residue preceding the proline. However, the enzyme activity assays illustrated that, TbPin1 displayed the phosphorylation-dependent isomerization ability for the substrate, but the FHA-truncated TbPar42 did not show the isomerase activity in the same experimental condition (Figure 5). Maybe some specific phosphorylated peptides or a peptide containing Glu- or Asp-Pro motif could serve as the substrate for TbPar42 [13], [34]. The results from the enzyme activity assays are consistent with the previous report that TbPin1 had the ability to rescue the lethal mitotic phenotype of a temperature-sensitive mutation in *Saccharomyces cerevisiae* while TbPar42 lacked the ability [30], suggesting that TbPin1 is a Pin1-type parvulin and TbPar42 is a non Pin1-type parvulin. TbPar42 shows well conserved residues to Pin1-type parvulins, but why it lacks the Pin1-like isomerase activity still remains to be elucidated.

The active site residues were originally defined by the crystal structure of hPin1 in complex with an Ala-Pro dipeptide [25], in which the dipeptide is used as a pseudo substrate for hPin1. In the present work, we identified the active site residues of TbPin1 for a phosphorylated peptide substrate using the chemical shift perturbation approach. NMR titration of the phosphorylated peptide to TbPin1 demonstrated that residues Ser15, Arg18, Asn19, Val21, Ser22, Val32, Gly66, Ser67, Met83, Asp105 and Gly107 experienced significant chemical shift changes (Figure 6). These residues may be involved in substantial contact with the phosphorylated peptide substrate. Most of these residue side chains point to the peptide binding pocket. For instance, in comparison with the corresponding residue Gln131 in hPin1, the Met83 side chain points in an orientation to the hydrophobic pocket (Figure S2). Dynamics data derived from the ^15^N relaxation experiments indicated that these residues except Gly66, Ser67 possessed significant conformational exchange rates R_ex_ on the µs-ms timescale (Figure 8). The result suggests that the substrate binding pocket is flexible, similarly with the previous result reported for the archaeal parvulin CsPinA [36].

On the other hand, the crucial residue Cys65 did not exhibit marked chemical shift perturbation during the peptide titration process, even though the PPIase activity analysis demonstrated that the mutation of Cys65 to Ala abolished the enzyme activity of TbPin1. It is well known that chemical shift perturbations monitored by 2D 1H-15N HSQC spectrum are sensitive to the binding of backbone amide groups, but usually not sensitive to that of side-chain groups. This reason might account for why the residues which were considered crucial for enzyme catalysis (including His9, Lys13, Cys65, Leu74, Met82, Phe86, Ser106 and His109 in TbPin1, equivalent to His59, Lys63, Cys113, Leu122, Met130, Phe134, Ser154 and His157 in hPin1) did not experience significant chemical shift perturbations [13]. Similarly, His12, Lys16, Cys70, Leu79, and His114 in PinAt (equivalent to His9, Lys13, Cys65, Leu74 and His109 in TbPin1) also showed non-significant chemical shift perturbations in 2D ^1^H-^15^N HSQC spectrum [43]. However, in 2D ^1^H-^13^C HSQC spectrum, the two conserved histidine residues (i.e. His9 and His86 in CsPinA, His146 and His239 in *S.aureus* PrsA-PPIase) displayed significant chemical shift perturbations [34], [36]. It was postulated that a hydrogen bonding between the two histidine residues were required for the catalysis [34], [42]. Therefore, both His9 and His109 in TbPin1 might also be involved in the catalysis.

Although TbPin1 exhibited evident phosphorylation-dependent PPIase activity for the phosphorylated peptide, chemical shift perturbation analysis illustrated that TbPin1 interacted weekly with the phosphorylated peptide. The weak binding affinity of TbPin1 for the substrate is supported by the previous studies conducted on other parvulins [34], [55], [56], which might be due to the fast conformational exchange between the free and complex forms.

In conclusion, the solution structure of TbPin1 from *Trypanosoma brucei* was determined and characterized using NMR spectroscopy. TbPin1 only contains a unique PPIase domain and adopts the typical catalytic tertiary structure (β-α2-η-β-α-β2 fold) of Pin1-type parvulins. The overall structure of TbPin1 is highly rigid except that the substrate binding pocket is flexible. TbPin1 catalyzes the *cis/trans* conformational change of the phosphorylated peptide {SSYFSG[p]TPLEDDSD}, and residues including Ser15, Arg18, Asn19, Val21, Ser22, Val32, Gly66, Ser67, Met83, Asp105 and Gly107 are involved in the substantial contact with the phosphorylated substrate. Mutation of Cys65 to Ala confirms that the conserved Cys is crucial for the enzyme catalysis in Pin1-type parvulins. These results shed light on the molecular mechanism of enzyme catalysis for TbPin1 and other Pin1-type parvulins.

## Materials and Methods

### Sample preparation

The amplified gene fragments coding for TbPin1 and TbPPIase (truncation of FHA domain of TbPar42) were cloned into the vector pET28b via NdeI and SalI restriction sites as mentioned previously [30]. A single point mutant on the pET28b-TbPin1 plasmid (C65A) was constructed using the site-directed mutagenesis. The recombinant proteins were expressed at 37°C in *E.coli* strain BL21 (DE3) cells and induced with 0.4 mM IPTG for 4 hours in LB media until an OD600 of ∼1.0. For ^15^N-labeled or ^15^N,^13^C-labeled TbPin1 protein samples, cells were grown in a M9 minimal medium containing ^15^N-NH_4_Cl (Cambridge Isotopes Laboratories) or ^15^N-NH_4_Cl and ^13^C_6_-Glucose (Cambridge Isotopes Laboratories) as sole nitrogen or/and carbon sources, respectively. Cells were resuspended in a lysis buffer containing 20 mM sodium phosphate (pH 7.4), 500 mM NaCl, 10 mM β-mercaptoethanol, 1% (V/V) Tween 20 and sonicated at 4°C. Following sonication, soluble supernatants were incubated with a Ni-NTA resin (Qiagen) for 2 hours and then washed with a lysis buffer without Tween 20 and a wash buffer containing 20 mM sodium phosphate (pH 7.4), 500 mM NaCl, 20 mM imidazole. The recombinant proteins were eluted with a wash buffer supplemented with 500 mM imidazole. Eluted proteins were digested with thrombin (Sigma) overnight at 4°C and followed by FPLC gel-filtration on a Superdex 75 10/300 GL column (GE Healthcare). Three additional residues (Gly-Ser-His) were remained at the N terminus of TbPin1 after thrombin cleavage and were not included in later assignment and structure calculation. The resulting samples were concentrated to 0.8∼1 mM and then exchanged into 20 mM sodium phosphate buffer (pH 7.0), 1 mM DTT, 0.01 mM NaN_3_.

Both phosphopeptide {SSYFSG[p]TPLEDDSD} and non-phosphorylated peptide SSYFSGTPLEDDSD were purchased from ChinaPeptides Corporation (Shanghai, China). Concentrated peptide stocks were prepared in 20 mM sodium phosphate buffer (pH 7.0).

### NMR Spectroscopy and Resonance Assignment

All NMR spectra were recorded at 298 K on a Varian Unity Inova 600 MHz spectrometer equipped with three RF channels and a triple-resonance pulse-field gradient probe. Sequence-specific assignments of backbone ^1^H, ^13^C, and ^15^N resonances were performed by using a suit of 3D heteronuclear spectra including HNCACB, CBCA(CO)NH, HNCA, HN(CO)CA, HNCO, HN(CA)CO, and HAHB(CO)NH spectra. Side-chain assignments were obtained by using 3D H(CCH)(CO)NHTOCSY, (H)CC(CO)NHTOCSY, HCCH-TOCSY, CCHTOCSY and ^15^N-edited TOCSY spectra. Secondary structures were predicted from secondary chemical shifts of ^1^H_α_, ^13^C_α_, ^13^C_β_ and ^13^CO with the Chemical Shift Index (CSI) approach [57]. A mixing time of 100 ms was used for 3D ^15^N- and ^13^C-edited NOESY experiments. All NMR data were processed with NMRPipe [58] and analyzed by using Sparky (T. D. Goddard and D. G. Kneller, University of California, USA) and CcpNmr Analysis [59].

### Structure Calculation

Conformational restraints were used for structure calculation of TbPin1, including ^1^H-^1^H distance restraints derived from 3D ^15^N- and ^13^C-edited NOESY spectra, backbone dihedral angle restraints generated by the TALOS+ software [41], and hydrogen bonds restraints derived from H-D exchange experiments. Structures were calculated using the ARIA2.2/CNS1.2 software [39], [40]. A family of 200 structures was calculated according to the simulated annealing protocol. Twenty lowest energy structures were selected and further refined in water. Quality of the ensemble was assessed by the PROCHECK program [60], [61]. Figures were generated with MOLMOL [62] or PyMOL (DeLano Scientific LLC).

### Enzyme activity analysis

NMR experiments were performed on peptide samples in a NMR buffer containing 20 mM sodium phosphate, 90% H_2_O and 10% D_2_O (pH 7.0) with or without TbPin1, the TbPin1-C65A mutant or the FHA-truncated TbPar42 mutant. The final concentration of the phosphorylated peptide {SSYFSG[p]TPLEDDSD} or non-phosphorylated peptide SSYFSGTPLEDDSD was 2.4 mM, and the final concentration of the protein was 0.03 mM. 2D ^1^H-^1^H NMR spectra were recorded with spectral widths of 8000×8000 Hz in t_1_ × t_2_ dimensions. EXSY spectra were acquired at a mixing time of 200 or 300 ms with 16 scans, while TOCSY spectra were recorded at a mixing time of 75 ms with 16 scans [22], [30], [46].

### Chemical shift perturbation

^15^N-labeled TbPin1 was dissolved in the NMR buffer described above. The phosphorylated peptide was titrated stepwise up to a final molar ratio of 1∶8, with each step monitored by recording 2D ^1^H-^15^N HSQC spectrum. The reciprocal titration of ^15^N-labeled TbPin1 with the phosphorylated peptide was performed accordingly with the initial TbPin1 concentration of 0.4 mM. The chemical shift changes of amide ^1^H and ^15^N chemical shifts were calculated by using Eq.1.
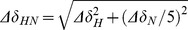
(1)


### ^15^N Relaxation Measurements

All ^15^N relaxation data were recorded at 298 K on Varian Unity Inova 600 spectrometer. The standard pulse sequences with minimal water suppression were used to record the 2D spectra of T_1_, T_2_, and {^1^H}-^15^N NOE. In the direct (^1^H) dimension, the carrier frequency was set on the water resonance with a spectral width of 10,000 Hz; while in the indirect (^15^N) dimension, the spectral width was 1400 Hz. A recycle delay time was 2 s. T_1_ was measured by using a series of spectra recorded with 10 relaxation delays: 10.83, 54.17, 108.34, 216.68, 325.02, 541.70, 866.72, 1191.74, 1570.93, 1950.12 ms. T_2_ measurement was carried out with 10 relaxation delays: 15.62, 31.23, 46.85, 62.46, 78.08, 93.70, 109.31, 124.93, 140.54 and 156.16 ms. The relaxation rates and experimental errors were calculated by a mono-exponential curve fitting of the corresponding signals in a series of 2D spectra using the CCPN software [59]. The steady-state {^1^H}-^15^N NOE enhancements were determined from the ratio of peak heights in spectra recorded with or without proton saturation. The saturated spectra were recorded with a 2 s relaxation delay followed by a 3 s period of proton saturation. The spectra without proton saturation were acquired with a relaxation delay of 5 s. The experimental errors in NOE values can be assessed as follows [63]:
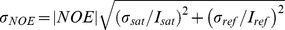
(2)where I and σ denote the peak intensity and the level of experimental noise, respectively. In the saturation and non-saturation experiments NOE can be determined as follow [63]:
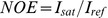
(3)


### Analysis of 15N relaxation data

Several methods have been developed for analyzing protein dynamics [51], [64], [65]. Here, we used the TENSOR2 program [48] and a suit of Mathematica notebooks provided by Spyracopoulos's lab [49] to analyze ^15^N relaxation data. TENSOR2 was used to determine rotational diffusion tensor from the coordinate PDB file and ^15^N relaxation data. TENSOR2 can also be used to perform the model-free analysis from relaxation rate R_1_, R_2_ and {1H}-^15^N NOE. Spyracopoulos et al. have developed a suit of Mathematica notebooks to analyze backbone ^15^N NMR relaxation data, such as spectral density, diffusion tensor, model-free analysis. These notebooks can be downloaded from www.bionmr.ulabert.ca/~lspy. The tensors and internal mobility parameters were estimated using Monte-Carlo sampling methods and F-tests [49].

## Supporting Information

Figure S1 **TbPin1 does not catalyze the** ***cis-trans*** **isomerization of the non-phosphorylated peptide.** A selected region of the 2D EXSY spectrum of the non-phosphorylated peptide SSYFSGTPLEDDSD is displayed (A) in the absence of TbPin1; (B) in the presence of TbPin1; (C) in the presence of the FHA-truncated TbPar42 mutant (TbPPIase). A mixing time of 300 ms was used. The *cis* and *trans* T7-HN and L9-HN are shown in the spectra, which were assigned based on the 2D-TOCSY spectrum of the peptide. No exchange cross peaks were observed in the spectra.
(EPS)

Figure S2 **Comparison of the binding sites between TbPin1 and hPin1.** (A) Binding sites of the phosphorylated peptide SSYFSG[p]TPLEDDSD on TbPin1 identified by using the chemical shift perturbation approach. (B) Binding sites of an Ala-Pro dipeptide and a sulfate ion on hPin1 which were defined by the crystal structure of hPin1 in complex with the dipeptide (PDB: 1PIN). These residues are highlighted in stick style and displayed in cyan and orange, respectively. The dipeptide and Q131 in hPin1 are colored magentas, slate, respectively. Q131 which is not considered to be involved in the dipeptide binding shows an orientation far away from the hydrophobic pocket constituted by H59, L122, M130, F134 and H157. However, the corresponding residue M83 in TbPin1 points in an orientation to the hydrophobic pocket, indicating that M83 is involved in the binding of the phosphorylated peptide.
(TIF)
