# Supplementary figures and images for: Solution Structural Analysis of the Single-Domain Parvulin TbPin1
Source: PLoS One. 2012 Aug 10;7(8):e43017. doi: 10.1371/journal.pone.0043017 (PMC3416822; doi:10.1371/journal.pone.0043017)

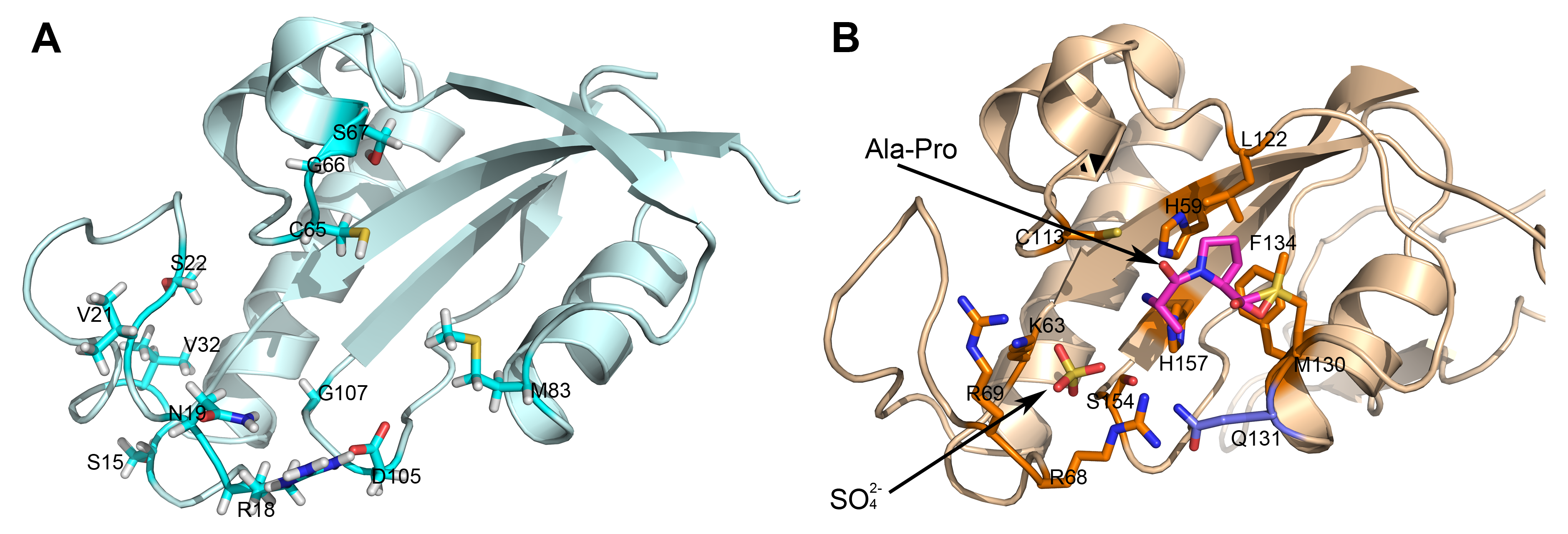

Supplement: Figure S2 — Comparison of the binding sites between TbPin1 and hPin1. (A) Binding sites of the phosphorylated peptide SSYFSG[p]TPLEDDSD on TbPin1 identified by using the chemical shift perturbation approach. (B) Binding sites of an Ala-Pro dipeptide and a sulfate ion on hPin1 which were defined by the crystal structure of hPin1 in complex with the dipeptide (PDB: 1PIN). These residues are highlighted in stick style and displayed in cyan and orange, respectively. The dipeptide and Q131 in hPin1 are colored magentas, slate, respectively. Q131 which is not considered to be involved in the dipeptide binding shows an orientation far away from the hydrophobic pocket constituted by H59, L122, M130, F134 and H157. However, the corresponding residue M83 in TbPin1 points in an orientation to the hydrophobic pocket, indicating that M83 is involved in the binding of the phosphorylated peptide. (TIF) [file pone.0043017.s002.tif]
